# Longitudinal hearing loss in Wolfram syndrome

**DOI:** 10.1186/s13023-018-0852-0

**Published:** 2018-06-27

**Authors:** Roanne Karzon, Anagha Narayanan, Ling Chen, Judith E. C. Lieu, Tamara Hershey

**Affiliations:** 1 Saint Louis Children’s Hospital, One Children’s Place, St. Louis, MO 63110 USA; 2 Program in Audiology and Communication Sciences, Washington University in St. Louis School of Medicine, St. Louis, MO 63110 USA; 3 Department of Psychiatry, Washington University in St. Louis School of Medicine, 4525 Scott Avenue, Campus Box 8134, St. Louis, MO 63110 USA; 4 Division of Biostatistics, Washington University in St. Louis School of Medicine, St. Louis, MO 63110 USA; 5 Department of Otolaryngology-Head and Neck Surgery, Washington University in St. Louis School of Medicine, St. Louis, MO 63110 USA; 6 Department of Radiology, Washington University in St. Louis School of Medicine, 4525 Scott Avenue, Campus Box 8134, St. Louis, MO 63110 USA

**Keywords:** Wolfram syndrome, Hearing loss, Speech intelligibility index

## Abstract

**Background:**

Wolfram syndrome (WFS) is a rare autosomal recessive disease with clinical manifestations of diabetes mellitus (DM), diabetes insipidus (DI), optic nerve atrophy (OA) and sensorineural hearing loss (SNHL). Although SNHL is a key symptom of WFS, there is limited information on its natural history using standardized measures. Such information is important for clinical care and determining its use as an outcome measure in clinical trials.

**Methods:**

Standardized audiologic measures, including pure-tone testing, tympanometry, speech perception, and the unaided Speech Intelligibility Index (SII) were assessed in patients with confirmed WFS annually. Mixed model analyses were used to examine main effects of age, time or interactions for pure tone average (PTA), high frequency average (HFA) and SII.

**Results:**

Forty WFS patients were evaluated between 1 and 6 times. Mean age at initial enrollment was 13.5 years (SD = 5.6). Patients were classified as having normal hearing (*n* = 10), mild-to-severe (*n* = 24) or profound SNHL (*n* = 6). Mean age of diagnosis for SNHL was 8.3 years (SD = 5.1) with 75% prevalence. HFA worsened over time for both ears, and SII worsened over time in the worse ear, with greater decline in both measures in younger patients. Average estimated change over 1 year for all measures was in the subclinical range and power analyses suggest that 100 patients would be needed per group (treatment vs. placebo) to detect a 60% reduction in annual change of HFA over 3 years. If trials focused on just those patients with SNHL, power estimates suggest 55 patients per group would be sufficient.

**Conclusions:**

Most patients had a slow progressive SNHL emerging in late childhood. Change over time with standard audiologic tests (HFA, SII) was small and would not be detectable for at least 2 years in an individual. Relatively large sample sizes would be necessary to detect significant impact on hearing progression in a clinical trial. Hearing function should be monitored clinically in WFS to provide appropriate intervention. Because SNHL can occur very early in WFS, audiologists and otolaryngologists should be aware of and refer for later emerging symptoms.

**Electronic supplementary material:**

The online version of this article (10.1186/s13023-018-0852-0) contains supplementary material, which is available to authorized users.

## Background

Wolfram syndrome (WFS) is an autosomal recessive neurodegenerative disease with an estimated prevalence worldwide from one in 55,000 to one in 770,000 [1–4]. The most frequently cited clinical manifestations include diabetes mellitus (DM), optic nerve atrophy (OA), diabetes insipidus (DI), sensorineural hearing loss (SNHL), neurological symptoms, renal tract abnormalities, psychiatric disorders and gonadal disorders [4, 5]. Minimal diagnostic criteria have often been reported as DM and OA [6–10]. Life expectancy can be reduced, particularly in patients with all of the classic clinical symptoms associated with the syndrome and when symptoms are not managed optimally [1, 6, 8, 11, 12].

The genetic cause for WFS has been described with pathogenesis attributed to mutations in the *WFS1* gene on chromosome 4p16.1 [13–16] and less commonly to *WFS2* (*CISD2*) [15]. Although there are currently no effective medical treatments, progress is being made using available medications and developing therapeutic agents to treat endoplasmic reticulum stress and dysfunction which underlie WFS as well as several other common diseases such as type 1 diabetes, type 2 diabetes and neurodegeneration [15–17].

The typical sequence of WFS has been described as DM followed by OA in the first decade with DI and SNHL potentially developing in later years [3, 6, 18–20]. Although the SNHL is typically high-frequency in nature, there are notable exceptions with respect to audiometric configuration [21]. There are now several reports of SNHL with early onset and presenting as the first symptom identified [9, 21–23]. Review of 392 analyzable patient records from 49 references indicated that the first WFS symptoms identified were as follows: DM, 79.50%; SNHL, 6.35%; OA, 17.08%; neurological, psychiatric developmental defects, 8.96%; and DI 2.70%, urological or renal defects, 5.26% [8].

There is growing appreciation for variability in the relative onset and severity of symptoms in WFS, which has implications for referring healthcare providers [4, 8, 9, 12, 21, 24–29]. With respect to hearing loss onset, many reports suggest that clinically significant hearing loss is usually diagnosed in the second or third decade [3, 6, 18, 19]. However, there are several reports of patients with early onset of hearing loss from birth to three years of age [9, 11, 12, 20–23, 30–34]. There are also reports of patients with SNHL onset in fourth decade and one patient with no hearing deficit as of 56 years of age [7, 9]. Early clinical reports were often case studies based on physical examination and observation by the patient or family. However, hearing loss can be under-reported if it is diagnosed based on patient or parent report rather than standardized audiometric measures [1, 3, 21, 35–39].

The majority of reports that include information regarding hearing in WFS do not include adequate descriptions of their methods or detail the type or severity of hearing loss. However, there are several notable exceptions [7, 21, 39]. Audiologic data from 9 Dutch patients with WFS included measures of air conduction (AC), bone conduction (BC), speech recognition and, for 2 patients, auditory brain stem response testing [7]. Data indicated progressive mid- and high-frequency SNHL, with “well-preserved speech recognition” for the degree of SNHL. Subsequently, no sex difference and no significant progression in hearing loss was reported for 23 patients with WFS assessed in a sound-treated room with AC and BC [39].

Accurate assessment of hearing in individuals with WFS is necessary to define this key symptom over the lifespan. The primary purpose of this study was to assess auditory status longitudinally in a relatively large cohort of 40 participants with WFS using a standardized test battery with commercially available instrumentation. The multidisciplinary nature of the Washington University Wolfram Syndrome Research Clinic afforded the opportunity to compare SNHL onset relative to the other cardinal WFS symptoms of DM, DI and OA.

## Methods

### Participants

WFS participants were recruited through direct or physician referral and the International Wolfram Syndrome Registry. Recruitment occurred in accordance with the approved procedures of the Human Research Protection Office of Washington University in Saint Louis. All participants provided informed consent prior to participation. Children under age 18 provided assent, and their parent or guardian provided written consent. In most cases the guardians or participants initiated joining the registry and thus were self-selected to participate in the research clinic. Participants were assessed during a multidisciplinary clinic held annually from 2010 to 2017. All participants were not tested each year.

### Procedures

Audiologic assessment was comprised of pure-tone testing, tympanometry, speech reception thresholds (SRTs) and speech discrimination testing in quiet and noise. Audiologic measures and calculations were conducted as previously described [21]. The minimum audiometric step size was 5 dB with threshold determination as described in the guidelines of the American Speech-Language and Hearing Association [40]. AC thresholds were obtained from 0.25 to 8 kHz. Inter-octave frequencies were assessed if there was 20 dB or greater difference between adjacent octaves. Hearing sensitivity was considered abnormal if the AC pure tone average (PTA) of 0.50, 1 and 2 kHz was > 20 dB hearing level (HL) or if more than 2 frequencies were > 20 dB HL. Hearing loss was defined as profound if the PTA was ≥80 dB HL. High frequency average (HFA) was calculated from 4 and 8 kHz thresholds. If the threshold at 8 kHz was beyond the audiometric limits, 95 dB HL was used for the calculation. BC was assessed if the AC threshold was ≥ 20 dB HL. In addition to direct test measures, the unaided SII was calculated for each ear. The unaided SII quantifies the proportion of speech information that is audible to the listener in a given setting. An SII of 0.0 (0%) suggests that no speech information is audible, and an SII of 1.0 (100%) indicates that all speech information in a given setting is audible [41–45]. As SII is often reported as a percentage in clinical applications, we have chosen to report percentages for our findings. An unaided SII less than 75% suggests significant compromise in speech transmission to the listener [46]. Tympanometry was included to assist in determining whether a conductive component was present. Speech testing was administered with recorded test stimuli. SRTs were obtained with the CID W1 spondee word list after familiarization. The BKB-SIN (Bamford-Kowal-Bench speech-in-noise test) [47] was delivered and scored per standard protocol.

### Instrumentation

Pure tone, SRT, and speech in noise testing (BKB-SIN) were conducted in a sound-treated examination room (Industrial Acoustics Company, Inc.). Stimuli were presented with a Madsen Orbiter-922, Grason Stadler Audiostar or 61 audiometer calibrated to American National Standards Institute (ANSI S3.6–1996) via sound field, insert earphones (ER-3A), or a bone conduction oscillator (Radioear B71). If excess cerumen precluded use of insert earphones, Telephonics TDH50 supraural headphones were used. Tympanometric measures of physical volume, static admittance and peak pressure were obtained with an Interacoustics AT235 or Grason Stadler tympanometer. The unaided SII was calculated on the Audioscan Verifit based on pure tone AC thresholds.

### Statistical approach

Our goal was to determine if any of the primary audiology metrics (HFA, PTA, SII) changed over time in patients with WFS, and whether the degree of change differed due to age or sex. We asked these questions for each ear separately. For each patient we determined which ear had worse hearing based on the unaided SII. If unaided SII was the same for both ears, the worse ear was determined by the HFA and, if necessary, PTA. Identical analyses were performed for worse and better ears separately, pooling data for all patients. In addition, as an exploratory analysis, we examined the same effects in the subset of patients with defined hearing loss at most recent hearing test.

Analyses assumed a linear relationship between the outcome variables (HFA, PTA, and unaided SII) and time and used a random slope model (mixed model) to predict average annual rate of change of the outcomes. This model allows slopes (annual rate of change) to vary randomly between patients and fits a separate regression line for each patient. Interaction between time and age at the first testing session was examined to determine if the average annual rate of change significantly differs with patient age at the first testing session. The estimated annual rate of change of the outcomes are plotted against patient age at the first testing session. All mixed model analyses were conducted using a two-sided test at a significance level of 0.05 using SAS 9.4 (SAS Institute, Cary, NC).

Using the estimated annual rate of change in audiologic metrics, we performed power analyses to determine the sample size necessary to detect a significant effect of an intervention. Extensive simulations were conducted to determine the sample size to achieve at least 80% power to detect a 50% or 60% reduction in mean annual rate of progression in the audiologic measure with the fastest progression at a significance level of 0.05. We assumed that a trial would measure the outcome every six months during a 3-year follow-up. Mean trajectories were simulated using the random slope model assuming mean annual rate of progression in placebo group estimated from the WFS group data and a random slope distributed N(0, δ^2^) and homoscedastic error distributed N(0, Ɛ^2^) with both δ^2^ and Ɛ^2^ estimated from the WFS group data. Means of audiologic measures were assumed to be equal at baseline for the two groups. We assumed equal allocation between the two groups (treatment vs. placebo) and 1000 simulated trials were analyzed with empirical power calculated.

## Results

### Participants (Table 1)

Five participants had cochlear implants and so could not be tested (1 male; 4 females, 7.6 to 14.0 years of age at enrollment). Thirty-five participants were tested at least once (17 males; 18 females, 5.1 to 25.8 years of age at enrollment). Data from year 1 of the 11 participants previously reported [21] are included in this dataset. Of the 35 patients tested, 35 had 1 or more sessions, 30 had 2 or more sessions, 26 had 3 or more, 18 had 4 or more sessions, 13 had 5 or more sessions and 6 had 6 sessions, not all in consecutive years.

**Table 1:** Clinical characteristics and age at onset of symptoms (years) for Wolfram syndrome patients (*n* = 40)

Patient number	Sex	Age at enrollment	Siblings	Diabetes mellitus	Diabetes insipidus	Hearing loss	Optic nerve atrophy	Number of analyzed sessions	Years between first and last session
WFS_01	M	12.9		3.5	9.0	9.0	5.0	1	–
WFS_02	F	10.9		6.2	9.7	Normal	9.5	6	4.95
WFS_03	M	17.9		4.6	6.0	6.0	6.0	6	4.95
WFS_04	F	23.7		2.2	12.3	5.0	12.3	6	4.94
WFS_05	F	13.7		3.9	No dx	1.7	12.6	Cochlear implant	–
WFS_07	M	7.3		2.7	7.3	11.2	7.0	5	3.95
WFS_09	M	14.4	WFS_10, 11	10.8	14.7	Normal	10.9	3	2.93
WFS_10	F	11.6	WFS_9, 11	7.0	11.0	Normal	8.0	5	3.95
WFS_11	M	8.3	WFS_9, 10	7.8	10.0	Normal	6.0	3	3.93
WFS_12	M	22.9		6.3	17.0	7.0	17.0	6	4.95
WFS_13	F	5.4		4.7	7.0	7.4	5.2	6	4.94
WFS_14	F	12.6		6.2	11.2	9.1	7.9	4	3.93
WFS_15	F	10.9		2.7	12.0	9.0	7.0	4	2.98
WFS_16	F	25.8		13.0	14.5	25.8	13.0	5	3.93
WFS_17	F	17.1	WFS_29	4.9	Not Diagnosed	16.3	15.3	5	3.93
WFS_18	M	12.0		5.1	10.3	11.9	10.1	4	2.97
WFS_19	F	11.9		Not Diagnosed	Not Diagnosed	3.0	5.7	Cochlear implant	–
WFS_22	M	15.7		13.9	Not Diagnosed	Normal	13.0	4	2.97
WFS_23	F	17.7	WFS_24, 25	5.0	Not Diagnosed	10.0	17.0	3	2.00
WFS_24	F	16.3	WFS_23, 25	4.0	5.5	8.5	14.0	3	2.00
WFS_25	F	7.9	WFS_23, 24	4.0	Not Diagnosed	7.9	7.0	2	2.00
WFS_27	M	10.1		3.8	8.9	10.1	8.0	3	2.00
WFS_28	M	6.9		3.8	Not Diagnosed	Normal	3.8	3	1.99
WFS_29	F	5.1	WFS_17	Not Diagnosed	Not Diagnosed	3.0	Not Diagnosed	2	1.00
WFS_30	F	22.7		6.9	22.4	Normal	19.6	2	1.00
WFS_31	F	10.8		4.5	Not Diagnosed	8.8	7.0	2	1.00
WFS_32	F	7.6		Not Diagnosed	Not Diagnosed	0.4	6.4	Cochlear implant	–
WFS_33	M	6.3		4.8	6.2	6.3	6.0	2	0.66
WFS_34	F	14.0	WFS_35	3.5	13.0	3.0	Not Diagnosed	1	–
WFS_35	F	17.1	WFS_34	3.5	6.2	6.0	13.0	2	0.60
WFS_36	F	11.1		7.0	10.1	6.1	10.0	1	–
WFS_37	F	21.8		6.0	17.0	16.0	18.0	1	–
WFS_38	M	12.9		Not Diagnosed	Not Diagnosed	2.8	10.9	Cochlear implant	–
WFS_39	M	13.0		3.0	Not Diagnosed	13.0	9.0	1	–
WFS_40	F	17.1		13.0	13.5	Normal	8.0	1	–
WFS_42	M	8.3		5.0	No dx	6.4	7.4	1	–
WFS_43	M	21.1		5.0	16.0	Normal	14.0	1	–
WFS_44	M	10.9		5.0	Not Diagnosed	7.0	9.0	1	–
WFS_45	M	6.0		4.0	Not Diagnosed	Normal	Not Diagnosed	1	–
WFS_46	M	21.2		5.0	Not Diagnosed	12.0	11.0	1	–
Summary statistics: # or mean (SD)	18 Males	13.5 (5.6)	4 Sibships	5.6 (2.9)	11.3 (4.2)	8.3 (5.1)	10.0 (4.0)	4 with cochlear implants	

Age at onset or diagnosis for SNHL relative to the onset of DM, DI and OA varied, with hearing loss as the first symptom in 7 and as the second symptom in 7 of the 40 participants. SNHL was found in 30 patients, for a prevalence of 75% and an average onset at 8.3 years. With respect to sex, 12 of 18 males (67%) and 18 of 22 females (82%) had SNHL. Sex distribution did not differ between those with SNHL and those without (Chi-sq = 1.2, *p* = .27).

**Fig. 1:**
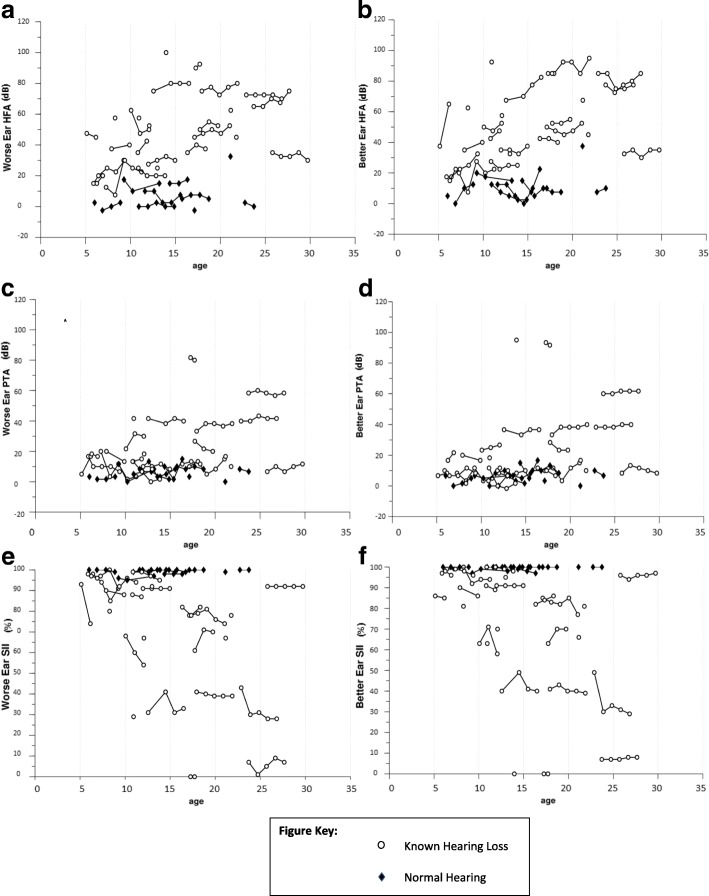
Scatter plots of raw data from each hearing measure (HFA, PTA and SII) for individuals with and without known SNHL. The worse ear data are in the left column and the better ear data are in the right column

Transient conductive hearing loss was present for 3 audiograms and 1 audiogram was judged to be invalid because it was inconsistent with prior and subsequent test results. These audiograms were excluded from statistical analysis. After these exclusions, we analyzed data from 35 patients with 1 or more sessions, 25 with 2 or more sessions, 19 with 3 or more, 13 with 4 or more sessions and 9 with 5 sessions. See Table 2 for group level performance on HFA, PTA and SII at session 1 (*n* = 35) and Fig. 1 and Additional file 1: Table S1 for raw audiologic data on all patients.

**Table 2:** Descriptive statistics for audiology variables at session 1 (*n* = 35)

	Mean	SD	Range
Worse Ear			
HFA (dB)	39	26	0–93
PTA (dB)	18	14	0–82
SII (%)	78	28	0–100
Better Ear			
HFA (dB)	38	27	−3–100
PTA (dB)	19	23	0–95
SII (%)	78	29	0–100

Based on case history and the most recent clinic hearing test, patients were placed in 3 hearing status categories: normal hearing (*n* = 10), mild-to-severe hearing loss (*n* = 24), and profound hearing loss of early onset (*n* = 6). Audiometric threshold data for octave frequencies from 250 to 8000 Hz may be found in Additional file 1: Table S1. These data include better ear results for all non-cochlear implant participants and the non-implanted ear of the participant with the unilateral cochlear implant. For this last category, SNHL was the first WFS symptom and was identified by 3 years of age. Of note, 5 participants reported no known hearing loss at their first evaluation, but were identified with SNHL through standardized hearing testing by this study.

Of the 10 participants with normal hearing on their most recent hearing test, 4 were beginning to show signs of impending hearing loss, with thresholds greater than 20 dB HL at 8 kHz. Of the remaining 6 participants, 2 were among the younger participants in the cohort: 6.0 and 8.9 years of age. Of note, one patient had completely normal AC responses from 250 Hz to 8 kHz and was one of the oldest participants in the cohort (age = 23.7 years). Twenty-seven participants were tested with the BKB-SIN. The other participants could not be tested due to cochlear implants (*n* = 5) and/or being non-native English speakers (*n* = 8). Administration of the BKB-SIN for the remaining 27 participants yielded SNR 50 results within normal limits for all but 2 participants who exhibited mildly reduced BKB-SIN performance that was within expectations for the magnitude of their hearing loss (unaided SII = 41 and 8%).

Of the 15 participants with an unaided SII less than 75% for the better ear, 14 use amplification devices (cochlear implants, *n* = 5; hearing aids, *n* = 8; frequency modulation device, *n* = 1). Six participants (15%) had SNHL by 3 years of age, with SNHL as the first symptom of WFS. This early onset included all 5 cochlear implant users.

### Statistical results

All the statistical results and inference from mixed-model analyses below included up to 5 time points since very few patients had observations at the sixth year testing session.

#### Whole group (Table 3)

##### Worse ear

**Fig. 2:**
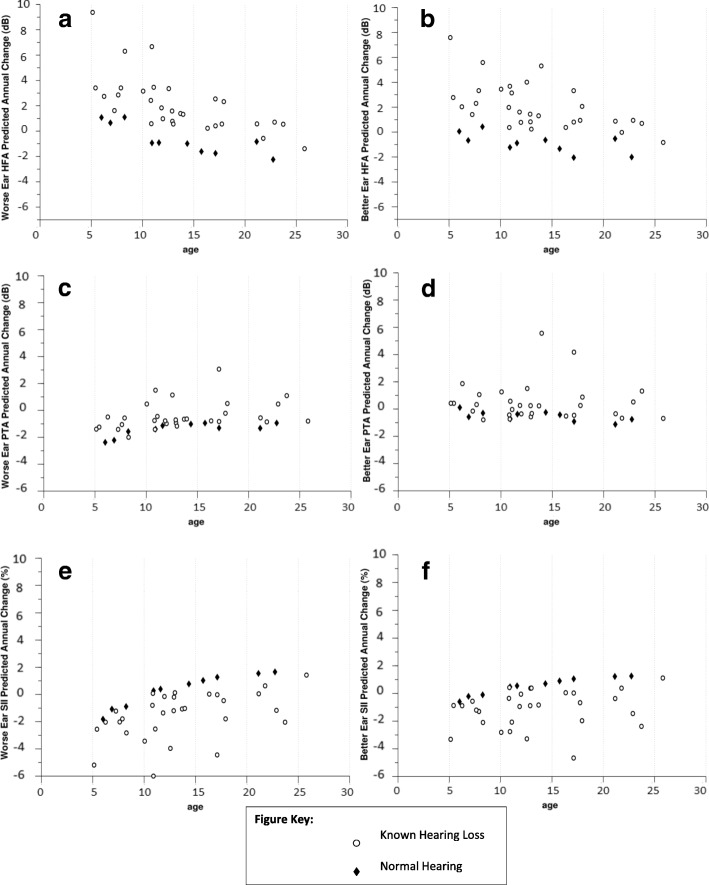
Estimated annual change in each hearing measure (HFA, PTA and SII) plotted over age at first testing session. The worse ear data are in the left column and the better ear data are in the right column

A main effect of time was found for HFA (*p* = .002) and SII (*p* = .01) with hearing becoming worse over time. No main effect of time was found for PTA (Fig. 1). Estimated average change in hearing over 1 year suggested minimal average annual decrease for all three measures (HFA = 1.77 dB increase; PTA = 0.09 dB increase; unaided SII = 1.50 percentage point decrease). Interactions were found between age at first session and time for HFA (*p* = .01) and unaided SII (*p* = .03), such that younger individuals tended to show more change in hearing than older individuals (Fig. 2).

**Table 3:** Results from Whole Group analyses, for worse and better ears (*n* = 35)

	HFA			PTA			SII		
	Time	Age	Time × age	Time	Age	Time × age	Time	Age	Time × age
Whole Group, Worse Ear									
p	0.002*	0.0003*	0.01*	0.09	0.43	0.24	0.01*	0.0002*	0.03*
F	11.54	14.97	7.52	3.01	0.64	1.42	8.31	15.58	4.86
Estimated average change over 1 year	1.77 dB increase			0.09 dB increase			1.50 decrease		
Whole Group, Better Ear									
p	0.01*	0.003*	0.05*	0.63	0.07	0.79	0.13	0.01*	0.38
F	7.71	9.81	4.00	0.23	3.38	0.07	2.41	7.70	0.78
Estimated average change over 1 year	1.64 dB increase			0.55 dB increase			1.22 decrease		

^*^ *p* < .05

##### Better ear

There was a main effect of time (*p* = .01) and borderline interaction between time and age at first session (*p* = .05) for HFA, but not for the other measures (Fig. 1).

#### Known hearing loss subset (Table 4)

##### Worse ear

There was a main effect of time for HFA (*p* = .001) and SII (*p* = .01) (Fig. 1), and an interaction between time and age at first session for HFA (*p* = .004) (Fig. 2). Estimated average change in hearing over 1 year was subclinical for HFA (2.37 dB increase) and SII (2.09 dB decrease).

**Table 4:** Results from known hearing loss subgroup analyses, for worse and better ears (*n* = 30)

	HFA			PTA			SII		
	Time	Age	Time × age	Time	Age	Time × age	Time	Age	Time × age
Known Hearing Loss, Worse Ear									
p	0.001*	0.0003*	0.004*	0.10	0.51	0.18	0.01*	0.001*	0.06
F	16.72	15.12	9.47	2.88	0.45	1.82	7.59	12.75	3.68
Estimated average change over 1 year	2.37 dB increase			0.04 dB decrease			2.09 decrease		
Known Hearing Loss, Better Ear									
p	0.003*	0.01*	0.03*	0.88	0.09	0.90	0.14	0.01*	0.47
F	10.57	8.07	4.81	0.02	3.02	0.02	2.36	7.21	0.54
Estimated average change over 1 year	2.17 dB increase			0.62 dB increase			1.72 decrease		

^*^ *p* < .05

##### Better ear

There was a main effect of time (*p* = .003) and an interaction between time and age at first session (*p* = .03) for HFA. The estimated change over 1 year was a 2.17 dB increase for HFA.

#### Effects of sex

There were no main effects of sex or interactions between sex and session for any of the three measures for either ear or for either group (*p* > .19).

#### Power analyses

Simulations were based on estimated annual rate of change in worse ear HFA for the entire WFS group and for those with SNHL. We ran simulations to determine sample sizes necessary to detect a 50% or 60% reduction in annual rate of change with 80% or greater power at a significance level of 0.05, assuming that testing occurred every 6 months from baseline to 3 years after treatment. For the entire group, we estimate that it would require 150 WFS patients per group (treatment vs placebo) to detect a 50% change and 100 WFS patients per group to detect a 60% slowing of progression of HFA. For the subgroup with SNHL, we estimate that it would take 75 WFS patients per group to detect a 50% change and 55 patients per group to detect a 60% slowing of progression of HFA.

## Discussion

We examined prevalence, age of onset, severity and quantitative progression for SNHL in children, adolescents and young adults with WFS. In this relatively young cohort, we found that the mean SNHL diagnosis was 8.3 years (SD = 5.10) with 75% prevalence. Although there was substantial individual variability across our cohort (Fig. 1), on average, HFA and unaided SII worsened over time and these effects were somewhat greater in younger patients. The quantitative annual changes observed were small, with average estimated change over 1 year in the subclinical range (less than 5 dB). Within clinical trials lasting 3 years, HFA may be the most useful audiologic metric, particularly for patients with known SNHL at the start of the trial. However, even for this metric and selected subgroup, simulations suggested that at least 55 WFS patients would be needed per group (treatment vs. placebo) to detect a 60% reduction in progression, which may be challenging given the low prevalence of WFS. Because hearing loss can occur very early in WFS, audiologists and otolaryngologists should be aware of this syndrome and refer patients as appropriate for consultation if other WFS-related symptoms emerge.

Prevalence of SNHL within our cohort was 75% which is within the range reported by other centers, despite the fact that our cohort is relatively young. Notably, of the 10 participants who exhibited normal hearing, 4 had reduced thresholds at 8 kHz suggesting that they may develop operationally defined hearing loss in the future. Due to our relatively young cohort, we cannot comment on the possibility that some patients with WFS may maintain normal hearing or develop only slight hearing impairment even into mid to late adulthood [7, 9].

Within our cohort three categories of hearing sensitivity were observed: normal hearing (*n* = 10), mild-to-severe SNHL (*n* = 24) and profound SNHL (*n* = 6). Because no universally accepted classification scheme for hearing loss could be identified, we provided the threshold data for individual participants in Additional file 1: Table S1. These data allow classification according to the scale of choice for each clinic. More importantly, these data allow direct comparison of hearing function for participants across clinical research studies. If researchers use calibrated equipment and trained personnel to administer hearing tests, reporting threshold data and uploading it to an international registry will lead more quickly to a better understanding of hearing loss progression in WFS.

The average age of SNHL onset for our cohort was 8.3 years, which is younger than most previous reports. Plausible explanations for this difference include differences in diagnostic criteria, age of the cohort and variable expression or penetrance of the mutated gene. Adding to reports of early-onset SNHL [9, 11, 12, 20–23, 29–33], 6 of the 30 patients (20%) with SNHL had severe-to-profound hearing loss with onset by 3 years of age.

It is likely that periodic audiologic evaluation, rather than relying on patient or parent report, increases sensitivity for early identification of SNHL. In support of this explanation, 5 participants thought to have normal hearing based on case history were identified with SNHL at an annual Washington University Wolfram Syndrome Research Clinic. Another factor is our diagnostic criteria for SNHL of greater than 20 dB HL for the PTA or 2 or more test frequencies worse than 20 dB HL, which may be stricter than those used by others.

Examining relative onset of SNHL to DM and OA in our cohort of 40 participants, SNHL was the first symptom identified for 7 participants (18%) and the second symptom identified for 7 participants (25%) This percentage of SNHL as the first symptom is higher than the 6.4% (first symptom) reported for a much larger cohort of 392 patients with WFS [8]. However, our percentage of 18% (second symptom) is similar to the 16.4% reported in the larger cohort [8]. It is not likely that diagnostic criteria account for this difference as 6 of the 7 participants in the current study, for whom SNHL was the first symptom, had severe-to-profound SNHL by 3 years of age.

With the exception of early onset SNHL, progression of hearing loss in WFS appears to occur slowly. Although HFA and unaided SII worsened over time numerically on average, progression would be clinically undetectable for several years. For example, within the entire cohort, there was an estimated average 1.77 dB change over 1 year in HFA in the worse ear (Table 3). Within the subset of patients with known hearing loss, this estimated average change was slightly larger with 2.37 dB per year for the worse ear, but was still in the subclinical range (Table 4). Since the standard audiometric step size is 5 dB, it may take almost 3 years to observe a clinically significant change in hearing at the group level, although individuals may differ. Our results also suggest that younger patients may change slightly faster than older patients. This effect was somewhat unexpected and could be explained by patients having a larger audiometric dynamic range at early ages or because of our restricted sampling at the older ages (Fig. 1). Although rate of progression has been previously reported [7, 38], differences in the age of the cohorts and statistical methods preclude definitive comparison. Larger sample sizes with a wider age range are necessary to confirm our observations.

With regard to amplification, we explored the role of the unaided SII as a possible guide for counseling. Fourteen of the 15 patients with SNHL and unaided SIIs less than 75% routinely used amplification. In clinical practice, the recommendation for a hearing aid evaluation would be based on the unaided SII in conjunction with the audiogram (PTA, HFA) and speech perception results. Further investigation of the unaided SII as a guideline for amplification candidacy in this population is needed.

Strengths of our study include use of standardized audiologic procedures with calibrated commercially available equipment and test administration by experienced, licensed audiologists. Inclusion of a speech in noise test (BKB-SIN) and calculation of the unaided SII allowed us to examine hearing care and treatment options for patients with WFS. The use of experienced audiologists facilitated diagnosis of conductive hearing loss and resolution of the one case involving inconsistent test results. In addition, few studies of hearing with WFS have repeated hearing measures over several years for many participants. Limitations of the current study include use of a small sample size and lack of representation at the older ages. In addition, selection criteria may have biased the sample because participants had to have the interest and ability to attend the WFS clinic.

To more fully elucidate hearing loss trajectories for WFS, it would be necessary to pool longitudinal data across clinics and countries [8, 9, 12, 48]. To ensure valid conclusions it is imperative that standardized test procedures, such as those reported in the current study, be used and that the definition of hearing loss be agreed upon among research groups [8, 48]. A suggested minimal test battery to determine severity and type of hearing loss would be AC, BC and tympanometry, administered by trained and experienced audiologists. Suggestions for an expanded test battery to plan intervention include speech perception tests (in quiet and in noise), calculation of the SII and possibly a quality of life questionnaire.

Early diagnosis of WFS allows for appropriate counseling and treatment to improve quality of life for patients with WFS. In concordance with other recent reports, both the presence and onset of the more frequent symptoms of WFS (DM, OA, and SNHL) are quite variable. Therefore, it is important for health care professionals who examine and diagnose diabetes, hearing loss and visual impairment to consider referral for genetic assessment of WFS when more than one symptom appears and/or family history includes individuals with more than one of the symptoms associated with WFS. With respect to hearing loss, it is best practice to make sure that vision is examined and corrected to the extent possible, as these patients may rely more heavily on visual cues to compensate for hearing loss.

## Conclusions

Accurate identification of the type and severity of SNHL in patients with WFS requires periodic audiologic assessment with a standardized test battery administered by qualified personnel from the onset of WFS diagnosis. SNHL is prevalent in WFS and preliminary results suggest that in addition to routine audiologic measures, the unaided SII may be a useful measure for determining amplification candidacy. Patients with WFS who acquired SNHL beyond early childhood experienced a slow progression on an annual basis. Our results suggest that HFA may be the most useful metric of change in hearing, but only over long periods of time (e.g., 3 or more years) and with relatively large sample sizes, making its use in clinical trials for a rare disorder such as WFS challenging. It is likely that other measures (e.g., visual acuity) may be more sensitive and thus require fewer patients to adequately power clinical trials. However, since SNHL may be the first symptom in some patients with WFS, hearing tests should be administered when patients present with a concomitant symptom such as DM or OA.

## Additional file

Additional file 1: **Table S1.** Raw data for the better ear for each participant and time point. (DOCX 43 kb)

## Abbreviations

AC: Air conduction
BC: Bone conduction
DI: Diabetes insipidus
DM: Diabetes mellitus
HFA: High frequency average
HL: Hearing level
OA: Optic nerve atrophy
PTA: Pure tone average
SII: Speech Intelligibility Index
SNHL: Sensorineural hearing loss
WFS: Wolfram syndrome

## References

[1] Barrett TG, Bundy SE (1997). Wolfram (DIDMOAD) syndrome. J Med Genet.

[2] Ganie MA, Bhat D (2009). Current developments in Wolfram syndrome. J Pediatr Endocrinol Metab.

[3] Kumar S (2010). Wolfram syndrome: important implications for pediatricians and pediatric endocrinologists. Pedatr Diabetes.

[4] Lombardo F, Salzano G, Di Bella C, Aversa T, Pugliatti F, Cara S, Valenzise M, De Luca F, Rigoli L (2014). Phenotypical and genotypical expression of Wolfram syndrome in 12 patients from a Sicilian district where this syndrome might not be so infrequent as generally expected. J Endocrinol Investig.

[5] Marshall BA, Permutt MA, Paciorkowski AR, Hoekel J, Karzon R, Wasson J, Viehover A, White NH, Shimony JS, Manwaring L, Austin P (2013). Phenotypic characteristics of early Wolfram syndrome. Orphanet J Rare Dis.

[6] Barrett TG, Bundy SE, Macleod AF (1995). Neurodegeneration and diabetes: UK nationwide study of Wolfram (DIDMOAD) syndrome. Lancet.

[7] Pennings RJ, Huygen PL, van den Ouweland JM, Cryns K, Dikkeschei LD, Van Camp G, Cremers CW (2004). Sex-related hearing impairment in Wolfram syndrome patients identified by inactivating WFS1 mutations. Audiol Neurootol.

[8] de Heredia ML, Cleries Soler R, Nunes Martinez V (2013). Genotypic classification of patients with Wolfram syndrome: insights into the natural history of the disease and correlation with phenotype. Genet Med.

[9] Chaussenot A, Rouzier C, Quere M, Plutino M, Ait-El-Mkadem S, Bannwarth S, Barth M, Dollfus H, Charles P, Nicolino M, Chabrol B (2015). Mutation update and uncommon phenotypes in a French cohort of 96 patients with WFS1-related disorders. Clin Genet.

[10] Paris LP, Usui Y, Serino J, Sá J, Friedlander M (2015). A challenging form of non-autoimmune insulin-dependent diabetes in a Wolfram syndrome patient with a novel sequence variant. J Diabetes Metab.

[11] Kinsley BT, Swift M, Dumont RH, Swift RG (1995). Morbidity and mortality in the Wolfram syndrome. Diabetes Care.

[12] Matsunaga K, Tanabe K, Inoue H, Okuya S, Ohta Y, Akiyama M, Taguchi A, Kora Y, Okayama N, Yamada Y, et al. Wolfram syndrome in the Japanese population; molecular analysis of WFS1 gene and characterization of clinical features. PLoS ONE. 2014;9: 10.1371/journal.pone.0106906. 10.1371/journal.pone.0106906 PMC4161373 25211237

[13] Inoue H, Tanizawa Y, Wasson J, Behn P, Kalidas K, Bernal-Mizrachi E, Mueckler M, Marshall H, Donis-Keller H, Crock P (1998). A gene encoding a transmembrane protein is mutated in patients with diabetes mellitus and optic atrophy (Wolfram syndrome). Nat Genet.

[14] Strom TM, Hörtnagel K, Hofmann S, Gekeler F, Scharfe C, Rabl W, Gerbitz KD, Meitinger T (1998). Diabetes insipidus, diabetes mellitus, optic atrophy and deafness (DIDMOAD) caused by mutations in a novel gene (Wolframin) coding for a predicted transmembrane protein. Hum Mol Genet.

[15] Urano F (2016). Wolfram syndrome: diagnosis, management, and treatment. Curr Diab Rep.

[16] Ariyasu D, Yoshida H, Hasegawa Y (2017). Endoplasmic reticulum (ER) stress and endocrine disorders. Int J Mol Sci.

[17] Danielpur L, Sohn YS, Karmi O, Fogel C, Zinger A, Abu-Libdeh A, Israeli T, Riahi Y, Pappo O, Birk R (2016). GLP-1-RA corrects mitochondrial labile iron accumulation and improves β-cell function in type 2 Wolfram syndrome. J Clin Endocrinol Metab.

[18] Medlej R, Wasson J, Baz P, Azar S, Salti I, Loiselet J, Permutt A, Halaby G (2004). Diabetes mellitus and optic atrophy: a study of Wolfram syndrome in the Lebanese population. J Clin Endocrinol Metab.

[19] Domenech E, Gómez-Zaera M, Nunes V (2004). Study of the WFS1 gene and mitochondrial DNA in Spanish Wolfram syndrome families. Clin Genet.

[20] Aloi C, Salina A, Pasquali L, Lugani F, Perri K, Russo C, Tallone R, Ghiggeri GM, Lorini R, d’Annunzio G (2012). Wolfram syndrome: new mutations, different phenotype. PLoS One.

[21] Karzon RK, Hullar TE (2013). Audiologic and vestibular findings in Wolfram syndrome. Ear Hear.

[22] Smith CJ, Crock PA, King BR, Meldrum CJ, Scott RJ (2004). Phenotype-genotype correlations in a series of wolfram syndrome families. Diabetes Care.

[23] Hansen L, Eiberg H, Barrett T, Bek T, Kjærsgaard P, Tranebjærg L, Rosenberg T (2005). Mutation analysis of the WFS1 gene in seven Danish Wolfram syndrome families; four new mutations identified. Eur J Hum Genet.

[24] Rendtorff ND, Lodahl M, Boulahbel H, Johansen IR, Pandya A, Welch KO, Norris VW, Arnos KS, Bitner-Glindzicz M, Emery SB, Mets MB (2011). Identification of p. A684V missense mutation in the WFS1 gene as a frequent cause of autosomal dominant optic atrophy and hearing impairment. Am J Med Genet A.

[25] Lieber DS, Vafai SB, Horton LC, Slate NG, Liu S, Borowsky ML, Calvo SE, Schmahmann JD, Mootha VK (2012). Atypical case of Wolfram syndrome revealed through targeted exome sequencing in a patient with suspected mitochondrial disease. BMC Med Genet..

[26] Bonnycastle LL, Chines PS, Hara T, Huyghe JR, Swift AJ, Heikinheimo P, Mahadevan J, Peltonen S, Huopio H, Nuutila P (2013). Autosomal dominant diabetes arising from a Wolfram syndrome 1 mutation. Diabetes.

[27] Sobhani M, Tabatabaiefar MA, Rajab A, Kajbafzadeh AM, Noori-Daloii MR. Significant expressivity of Wolfram syndrome: phenotypic assessment of two known and one novel mutation in the WFS1 gene in three Iranian families. Mol Biol Rep. 2014; 41(11):7499–505. 10.1007/s11033-014-3642-3 25173644

[28] Mozzillo E, Delvecchio M, Carella M, Grandone E, Palumbo P, Salina A, Aloi C, Buono P, Izzo A, D’Annunzio G (2014). A novel CISD2 intragenic deletion, optic neuropathy and platelet aggregation defect in Wolfram syndrome type 2. BMC Med Genet.

[29] Blanco-Aguirre ME, Rivera-De la Parra D, Tapia-Garcia H, Gonzalez-Rodriguez J, Welskin D, Arroyo-Yllanes ME, Escudero I, Nuñez-Hernandez JA, Medina-Bravo P, Zenteno JC (2015). Identification of unsuspected Wolfram syndrome cases through clinical assessment and WFS1 gene screening in type 1 diabetes mellitus patients. Gene.

[30] Marietti G, Bizzarri C, Perrone F, Zampino G, Conti G, Falsini B, Ricci B (1995). La sindrome di Wolfram. Presentazione di un caso clinico particolare. Minerva Pediatr.

[31] Giuliano F, Bannwarth S, Monnot S, Cano A, Chabrol B, Vialettes B, Delobel B, Paquis-Flucklinger V (2005). Wolfram syndrome in French population: characterization of novel mutations and polymorphisms in the WFS1 gene. Hum Mutat.

[32] Zalloua PA, Azar ST, Delépine M, Makhoul NJ, Blanc H, Sanyoura M, Lavergne A, Stankov K, Lemainque A, Baz P, Julier C (2008). WFS1 mutations are frequent monogenic causes of juvenile-onset diabetes mellitus in Lebanon. Hum Mol Genet.

[33] Mets RB, Emery SB, Lesperance MM, Mets MB (2010). Congenital cataracts in two siblings with Wolfram syndrome. Ophthalmic Genet.

[34] Nashibi M, Tajbakhsh A, Vahdati SM, Safari F, Mottaghi K (2016). DIDMOAD (Wolfram) syndrome. J Cell Mol Anesth.

[35] Gunn T, Bortolussi R, Little JM, Andermann F, Fraser FC, Belmonte MM (1976). Juvenile diabetes mellitus, optic atrophy, sensory nerve deafness, and diabetes insipidus—a syndrome. J Pediatr.

[36] Simsek E, Simsek T, TekgüT S, Hosal S, Seyrantepe V, Aktan G (2003). Wolfram (DIDMOAD) syndrome: a multidisciplinary clinical study in nine Turkish patients and review of the literature. Acta Paediatr.

[37] Lombardo F, Chiurazzi P, Hörtnagel K, Arrigo Τ, Valenzise Μ, Meitinger Τ, Messina ΜF, Salzano G, Barberi I, De Luca F (2005). Clinical picture, evolution and peculiar molecular findings in a very large pedigree with Wolfram syndrome. J Pediatr Endocrinol Metab.

[38] Homa K, Stefański A, Zmysłowska A, Molęda P, Bryśkiewicz ME, Majkowska L (2014). False diagnosis of type 1 diabetes mellitus and its complications in Wolfram syndrome—is it the reason for the low number of reported cases of this abnormality?. Endokrynol Pol.

[39] Plantinga RF, Pennings RJ, Huygen PL, Bruno R, Eller P, Barrett TG, Vialettes B, Paquis-Fluklinger V, Lombardo F, Cremers WR (2008). Hearing impairment in genotyped Wolfram syndrome patients. Ann Otol Rhinol Laryngol.

[40] American Speech-Language-Hearing Association. Guidelines for manual pure-tone threshold audiometry [Guidelines]. 2005. Available from https://www.asha.org/policy/GL2005-00014/.

[41] Hornsby BW (2004). The speech intelligibility index: what is it and what’s it good for?. Hear J.

[42] Scollie SD (2008). Children’s speech recognition scores: the speech intelligibility index and proficiency factors for age and hearing level. Ear Hear.

[43] Killion MC, Mueller HG (2010). Twenty years later: a new count-the-dots method. Hear J.

[44] Stiles DJ, Bentler RA, McGregor KK (2012). The speech intelligibility index and the pure-tone average as predictors of lexical ability in children fit with hearing aids. J Speech Lang Hear Res.

[45] Leal C, Marriage J, Vickers D (2016). Evaluating recommended audiometric changes to candidacy using the speech intelligibility index. Cochlear Implants Int.

[46] ANSI A (1997). S3. 5-1997, Methods for the calculation of the speech intelligibility index.

[47] Bench J, Kowal Å, Bamford J (1979). The BKB (Bamford-Kowal-Bench) sentence lists for partially- hearing children. Br J Audiol.

[48] Farmer A, Aymé S, de Heredia ML, Maffei P, McCafferty S, Młynarski W, Nunes V, Parkinson K, Paquis-Flucklinger V, Rohayem J (2013). EURO-WABB: an EU rare diseases registry for Wolfram syndrome, Alström syndrome and Bardet-Biedl syndrome. BMC Pediatr.

