# Supplementary material for: Longitudinal hearing loss in Wolfram syndrome
Source: Orphanet J Rare Dis. 2018 Jun 27;13:102. doi: 10.1186/s13023-018-0852-0 (PMC6020390; doi:10.1186/s13023-018-0852-0)
Supplement: Supplementary file 1 — Table S1. Raw data for the better ear for each participant and time point. (DOCX 43 kb) [file 13023_2018_852_MOESM1_ESM.docx]

**Table S1.** Raw data for the better ear for each participant and time point.

| **Study ID** | **Year data collected** | **Better Ear** | **Better Ear SII** | **Better Ear PTA** | **Better Ear .25k** | **Better Ear .5k** | **Better Ear 1k** | **Better Ear 2k** | **Better Ear 4k** | **Better Ear 8k** |
| --- | --- | --- | --- | --- | --- | --- | --- | --- | --- | --- |
| **WFS_01** | 2010 | L | 88 | 6.67 | 5 | 5 | 10 | 5 | 30 | 35 |
| **WFS_02** | 2010 | L | 100 | 3.33 | 10 | 0 | 0 | 0 | 0 | 0 |
|  | 2011 |  | 100 | 1.67 | 5 | 0 | 0 | 0 | 0 | 0 |
|  | 2012 |  | 100 | 8.33 | 10 | 10 | 5 | 5 | 5 | 0 |
|  | 2013 |  | 100 | 6.67 | 10 | 5 | 5 | 0 | 0 | 0 |
|  | 2014 |  | 100 | 5.00 | 10 | 5 | 0 | 0 | 0 | 0 |
|  | 2015 |  | 100 | 1.67 | 5 | 0 | 0 | 0 | 5 | 5 |
| **WFS_03** | 2010 | L | 41 | 18.33 | 20 | 15 | 20 | 65 | 80 | 70 |
|  | 2011 |  | 43 | 21.67 | 25 | 25 | 15 | 75 | 80 | 75 |
|  | 2012 |  | 40 | 23.33 | 25 | 25 | 20 | 70 | 80 | 65 |
|  | 2013 |  | 40 | 23.33 | 25 | 25 | 20 | 70 | 85 | 70 |
|  | 2014 |  | 39 | 23.33 | 20 | 25 | 25 | 70 | 85 | 75 |
|  | 2015 |  | 41 | 23.33 | 25 | 25 | 20 | 65 | 80 | 70 |
| **WFS_04** | 2010 | L | 7 | 55.00 | 45 | 50 | 70 | 60 | 60 | 70 |
|  | 2011 |  | 7 | 56.67 | 50 | 50 | 70 | 60 | 55 | 75 |
|  | 2012 |  | 7 | 58.33 | 50 | 50 | 75 | 60 | 60 | 80 |
|  | 2013 |  | 8 | 55.00 | 45 | 50 | 70 | 65 | 65 | 70 |
|  | 2014 |  | 8 | 55.00 | 45 | 50 | 70 | 65 | 70 | 80 |
|  | 2015 |  | 8 | 55.00 | 45 | 50 | 70 | 65 | 70 | 85 |
| **WFS_07** | 2010 | L | 99 | 11.67 | 15 | 15 | 5 | 5 | 5 | 20 |
|  | 2011 |  | 100 | 1.67 | 0 | 5 | 0 | 5 | 10 | 5 |
|  | 2012 |  | 92 | 15.00 | 15 | 15 | 15 | 5 | 5 | 55 |
|  | 2013 |  | 94 | 5.00 | 10 | 5 | 0 | 5 | 5 | 45 |
|  | 2014 |  | 94 | 6.67 | 10 | 5 | 5 | 5 | 0 | 45 |
| **WFS_09** | 2011 | R | 99 | 13.33 | 10 | 15 | 15 | 15 | 15 | 15 |
|  | 2012 |  | 98 | 6.67 | 10 | 10 | 0 | 5 | 10 | 20 |
|  | 2014 |  | 97 | 13.33 | 15 | 15 | 10 | 25 | 15 | 20 |
| **WFS_10** | 2010 | L | 100 | 10.00 | 15 | 10 | 5 | 10 | 10 | 10 |
|  | 2011 |  | 100 | 10.00 | 10 | 10 | 10 | 10 | 10 | 10 |
|  | 2012 |  | 100 | 10.00 | 15 | 10 | 5 | 5 | 5 | 0 |
|  | 2013 |  | 100 | 5.00 | 5 | 5 | 5 | 5 | 0 | 5 |
|  | 2014 |  | 100 | 8.33 | 10 | 10 | 5 | 10 | 5 | 10 |
| **WFS_11** | 2010* | L | 98 | 13.33 | 10 | 15 | 15 | 0 | 10 | 35 |
|  | 2011 |  | 97 | 10.00 | 15 | 10 | 5 | 5 | 10 | 25 |
|  | 2012 |  | 99 | 5.00 | 10 | 5 | 0 | 10 | 5 | 15 |
|  | 2013* |  | 94 | 15.00 | 15 | 15 | 15 | 5 | 20 | 40 |
|  | 2014* |  | 77 | 18.33 | 15 | 15 | 25 | 30 | 30 | 60 |
|  | 2015 |  | 98 | 6.67 | 10 | 5 | 5 | 10 | 10 | 20 |
| **WFS_12** | 2010 | L | 49 | 28.33 | 20 | 20 | 45 | 50 | 60 | 85 |
|  | 2011 |  | 30 | 25.00 | 10 | 20 | 45 | 50 | 60 | 85 |
|  | 2012 |  | 33 | 28.33 | 20 | 20 | 45 | 50 | 70 | 75 |
|  | 2013 |  | 31 | 30.00 | 20 | 25 | 45 | 50 | 70 | 75 |
|  | 2014 |  | 29 | 30.00 | 20 | 25 | 45 | 50 | 70 | 70 |
|  | 2015 |  | 23 | 35.00 | 25 | 30 | 50 | 55 | 75 | 75 |
| **WFS_13** | 2010 | R | 97 | 15.00 | 20 | 10 | 15 | 5 | 5 | 25 |
|  | 2011 |  | 99 | 8.33 | 10 | 10 | 5 | 5 | 10 | 30 |
|  | 2012 |  | 99 | 10.00 | 15 | 10 | 5 | 5 | 10 | 40 |
|  | 2013 |  | 98 | 13.33 | 15 | 15 | 10 | 10 | 10 | 35 |
|  | 2014 |  | 96 | 8.33 | 10 | 5 | 10 | 10 | 15 | 45 |
|  | 2015 |  | 92 | 13.33 | 20 | 10 | 10 | 10 | 20 | 50 |
| **WFS_14** | 2011 | R | 40 | 41.67 | 45 | 40 | 40 | 30 | 65 | 85 |
|  | 2013 |  | 49 | 38.33 | 40 | 40 | 35 | 25 | 65 | 95** |
|  | 2014 |  | 41 | 40.00 | 40 | 40 | 40 | 30 | 70 | 90 |
|  | 2015 |  | 40 | 41.67 | 50 | 40 | 35 | 35 | 70 | 90 |
| **WFS_15** | 2012 | L | 100 | 20.00 | 30 | 15 | 15 | 5 | 5 | 45 |
|  | 2013 |  | 100 | 6.67 | 20 | 0 | 0 | 0 | 0 | 40 |
|  | 2014 |  | 99 | 6.67 | 20 | 5 | -5 | -5 | 0 | 40 |
|  | 2015 |  | 98 | 6.67 | 15 | 5 | 0 | 0 | 0 | 40 |
| **WFS_16** | 2011 | R | 96 | 5.00 | 5 | 5 | 5 | 15 | 20 | 50 |
|  | 2012 |  | 94 | 10.00 | 10 | 10 | 10 | 20 | 25 | 40 |
|  | 2013 |  | 96 | 6.67 | 5 | 5 | 10 | 20 | 20 | 45 |
|  | 2014 |  | 96 | 8.33 | 10 | 5 | 10 | 15 | 20 | 50 |
|  | 2015 |  | 97 | 5.00 | 0 | 5 | 10 | 10 | 10 | 50 |
| **WFS_17** | 2011 | R | 85 | 6.67 | 5 | 10 | 5 | 15 | 35 | 55 |
|  | 2012 |  | 83 | 8.33 | 5 | 10 | 10 | 15 | 35 | 60 |
|  | 2013 |  | 82 | 5.00 | 5 | 5 | 5 | 0 | 40 | 60 |
|  | 2014 |  | 85 | 5.00 | 0 | 10 | 5 | 20 | 35 | 60 |
|  | 2015 |  | 77 | 13.33 | 15 | 10 | 15 | 20 | 40 | 65 |
| **WFS_18** | 2012 | L | 91 | 10.00 | 10 | 10 | 10 | 5 | 10 | 45 |
|  | 2013 |  | 91 | 8.33 | 10 | 5 | 10 | 5 | 15 | 45 |
|  | 2014 |  | 91 | 11.67 | 15 | 10 | 10 | 5 | 20 | 45 |
|  | 2015 |  | 91 | 13.33 | 15 | 15 | 10 | 5 | 15 | 45 |
| **WFS_22** | 2012 | R | 100 | 10.00 | 10 | 10 | 10 | 10 | 5 | 5 |
|  | 2013 |  | 100 | 11.67 | 15 | 5 | 15 | 10 | 5 | 10 |
|  | 2014 |  | 100 | 13.33 | 15 | 10 | 15 | 15 | 5 | 10 |
|  | 2015 |  | 100 | 6.67 | 5 | 5 | 10 | 10 | 5 | 5 |
| **WFS_23** | 2013 | R | 63 | 25.00 | 20 | 25 | 30 | 30 | 50 | 50 |
|  | 2014 |  | 70 | 21.67 | 20 | 20 | 25 | 25 | 50 | 60 |
|  | 2015 |  | 70 | 21.67 | 20 | 20 | 25 | 25 | 50 | 55 |
| **WFS_24** | 2013 | L | 82 | 13.33 | 15 | 15 | 10 | 10 | 35 | 35 |
|  | 2014 |  | 84 | 11.67 | 15 | 15 | 5 | 10 | 35 | 45 |
|  | 2015 |  | 86 | 6.67 | 10 | 10 | 0 | 10 | 35 | 40 |
| **WFS_25** | 2013 | R | 90 | 18.33 | 15 | 20 | 20 | 20 | 25 | 50 |
|  | 2014* |  | 40 | 33.33 | 30 | 30 | 40 | 45 | 55 | 70 |
|  | 2015 |  | 86 | 16.67 | 15 | 15 | 20 | 15 | 30 | 50 |
| **WFS_27** | 2013 | R | 63 | 21.67 | 25 | 20 | 20 | 30 | 60 | 65 |
|  | 2014 |  | 71 | 23.33 | 25 | 25 | 20 | 30 | 45 | 50 |
|  | 2015 |  | 58 | 20.00 | 20 | 20 | 20 | 40 | 50 | 50 |
| **WFS_28** | 2013 | R | 100 | 3.33 | 5 | 5 | 0 | -5 | 0 | -5 |
|  | 2014 |  | 100 | 5.00 | 10 | 5 | 0 | 0 | 0 | 0 |
|  | 2015 |  | 100 | 6.67 | 10 | 5 | 5 | 5 | 5 | 0 |
| **WFS_29** | 2014 | L | 86 | 8.33 | 10 | 10 | 5 | 5 | 35 | 60 |
|  | 2015 |  | 85 | 10.00 | 10 | 10 | 10 | 5 | 35 | 55 |
| **WFS_30** | 2014 | R | 100 | 8.33 | 5 | 10 | 10 | 10 | 5 | 0 |
|  | 2015 |  | 100 | 6.67 | 5 | 10 | 5 | 5 | 5 | -5 |
| **WFS_31** | 2014 | R | 91 | 10.00 | 20 | 10 | 0 | 0 | 10 | 60 |
|  | 2015 |  | 89 | 13.33 | 20 | 10 | 10 | 10 | 25 | 60 |
| **WFS_33** | 2014 | R | 98 | 25.00 | 35 | 25 | 15 | 10 | 10 | 20 |
|  | 2015 |  | 96 | 25.00 | 25 | 30 | 20 | 15 | 15 | 25 |
| **WFS_34** | 2014 | R | 0 | 80.00 | 55 | 80 | 105 | 100 | 105 | 95 |
|  | 2015 |  | 0 | 81.67 | 60 | 80 | 105 | 100 | 105 | 95 |
| **WFS_35** | 2014 | R | 0 | 80.00 | 60 | 80 | 100 | 100 | 85 | 95 |
|  | 2015 |  | 0 | 78.33 | 60 | 75 | 100 | 100 | 90 | 95 |
| **WFS_36** | 2015 | L | 70 | 15.00 | 25 | 10 | 10 | 15 | 55 | 50 |
| **WFS_37** | 2016 | R | 81 | 8.33 | 10 | 10 | 5 | 15 | 40 | 50 |
| **WFS_39** | 2016 | L | 95 | 8.33 | 5 | 5 | 15 | 10 | 10 | 40 |
| **WFS_40** | 2016 | L | 100 | 6.67 | 10 | 5 | 5 | 0 | 5 | -10 |
| **WFS_42** | 2016 | R | 81 | 3.33 | 5 | 5 | 0 | 0 | 50 | 65 |
| **WFS_43** | 2016 | R | 100 | 1.67 | 5 | 0 | 0 | 0 | 0 | 65 |
| **WFS_44** | 2017 | R | 63 | 6.67 | 5 | 5 | 10 | 40 | 45 | 70 |
| **WFS_45** | 2017 | R | 100 | 8.33 | 5 | 10 | 10 | 0 | 5 | 0 |
| **WFS_46** | 2017 | L | 66 | 10.00 | 10 | 10 | 10 | 30 | 55 | 70 |
|  |  |  |  |  |  |  |  |  |  |  |

* exclusion

** If no response at 90db @ 8k a value of 95db was assigned
